# Preoperative hydronephrosis predicts adverse pathological features and postoperative survival in patients with high-grade upper tract urothelial carcinoma

**DOI:** 10.1590/S1677-5538.IBJU.2020.0021

**Published:** 2020-11-18

**Authors:** Subo Qian, Chengcai Liang, Yu Ding, Chen Wang, Haibo Shen

**Affiliations:** 1 Shanghai Jiao Tong University School of Medicine Xinhua Hospital Shanghai China Department of Urology, Xinhua Hospital, School of Medicine, Shanghai Jiao Tong University, Shanghai, China; 2 Sun Yat-Sen University Cancer Center State Key Laboratory of Oncology in South China Department of Gastric and Pancreatic Surgery Guangzhou China Department of Gastric and Pancreatic Surgery, Sun Yat-Sen University Cancer Center, State Key Laboratory of Oncology in South China, Guangzhou, China

**Keywords:** Hydronephrosis, Nephroureterectomy, Urinary Tract

## Abstract

**Purpose:**

Epidemiological studies reported conflicting results about preoperative hydronephrosis in upper tract urothelial carcinoma (UTUC). This study aimed to investigate the association between preoperative hydronephrosis and pathologic features and oncologic outcomes in patients with UTUC treated by radical nephroureterectomy (RNU).

**Materials and Methods:**

This was a retrospective, single-center cohort study of 377 patients treated by RNU without perioperative chemotherapy between January 2001 and December 2014. Logistic regression, Cox regression, and survival analyses were performed.

**Results:**

Among the 226 patients with high-grade UTUC, 132 (58%) had preoperative hydronephrosis. Multivariable logistic regression revealed that hydronephrosis was independently associated with advanced pT stage (P=0.017) and lymph node or lymphovascular invasion (P=0.002). Median follow-up was 36 months (interquartile range: 20-48 months). The 3- and 5-year overall survival (OS) rates in patients with hydronephrosis were significantly lower than in those without hydronephrosis (both P <0.001). The 3- and 5-year cancer-specific survival (CSS) rates in patients with hydronephrosis were significantly lower than in those without hydronephrosis (both P=0.001). Hydronephrosis was independently associated with OS and CSS (P=0.001 and P=0.004, respectively). Among the 151 patients with low-grade UTUC, hydronephrosis was not associated with pathologic features and postoperative survival.

**Conclusions:**

Preoperative hydronephrosis was significantly associated with adverse pathologic features and postoperative survival in patients with high-grade UTUC.

## INTRODUCTION

Upper tract urothelial carcinoma (UTUC) is a rare malignancy, accounting for only 5%-10% of all urothelial carcinomas. UTUC arises from the urothelial lining of the urinary tract, from the renal calyces to the ureteral orifice, and the tumors of the renal pelvis and calyces are approximately twice as common as tumors of the ureters (1, 2). Although radical nephroureterectomy (RNU) is considered to be the preferred treatment for non-metastatic UTUC, disease progression often occurs and results in an unsatisfactory outcome, particularly in tumors with advanced pathologic stages (3-5). In addition, contemporary data suggested that patients with muscle-invasive (pT2 or greater) or non-organ-confined (pT3-4 or lymph node involvement [LNI]) UTUC may benefit from neoadjuvant chemotherapy (6, 7) and lymphadenectomy (8, 9). Therefore, adopting an accurate prediction for the pathologic features of the disease before surgery is of clinical significance for therapeutic planning.

Currently, conventional preoperative strategies for evaluating the pathologic features of UTUC are limited to radiographic imaging and biopsy (10). Unfortunately, these methods are not reliable enough, with low sensitivity and specificity. Uncovering surrogate predictors of pathologic features in UTUC is of clinical significance.

Hydronephrosis is a common comorbidity in the diagnosis of UTUC and is the result of malignant obstruction in the upper urinary tract. Interestingly, recent evidence demonstrated an association between hydronephrosis and adverse pathologic features or prognosis in UTUC (11-14), but the prognostic role of hydronephrosis was not fully supported by other reports (15-17). In addition, the study by Chung et al. showed that hydronephrosis, as a surrogate for adverse pathologic and oncologic features, was found only in high-grade UTUC patients (18).

Therefore, an identification of the predictive potential of hydronephrosis in UTUC would be of clinical significance. We hypothesized that hydronephrosis was associated with the pathologic features and prognosis of UTUC and that the associations might be different between low- and high-grade UTUC. The present study aimed to investigate the potential of hydronephrosis as a surrogate associated factor of pathologic features as well as oncologic outcomes.

## SUBJECTS AND METHODS

### Patients

The patients pathologically diagnosed with upper tract tumors and who underwent RNU at the Department of Urology of our hospital between January 2001 and December 2014 were retrospectively reviewed. The patients with UTUC and dominant urothelial cell histology were included. UTUC was pathologically confirmed by preoperative biopsy under ureteroscopy or by intraoperative frozen section. The exclusion criteria were: (1) another malignant disease within 5 years; (2) underwent conservative surgery; (3) previous or concomitant radical cystectomy; (4) metastatic diseases; (5) perioperative chemotherapy; or (6) incomplete medical data. This study was approved by the Institutional Review Board of our hospital (No. XHEC-D-2018-057). Informed consent was waived by the committee because of the retrospective nature of the study.

### Surgery

Patients underwent standard RNU (i.e., extrafascial dissection of the kidney with the entire ureter and adjacent segment of the bladder cuff). The hilar and regional lymph nodes adjacent to the ipsilateral great vessels were generally resected if palpable intraoperatively or if enlarged on preoperative axial imaging. All patients with previous or current non-muscle invasive bladder cancer (NMIBC) were treated by transurethral resection.

### Data collection

All postoperative specimens were histologically confirmed to be urothelial carcinomas by two pathologists. Baseline characteristics including age, gender, tumor side, size, location, previous or current NMIBC, history of hypertension or diabetes mellitus, primary tumor stage (pT), primary tumor grade (2), LNI, lymphovascular invasion (LVI), preoperative hydronephrosis, and type of surgery (open or laparoscopic) were collected. Hydronephrosis was defined as the dilation of the calyx or renal pelvis (≥1cm in the posterior-anterior plane), with or without renal parenchyma atrophy (19). Assessment of ipsilateral hydronephrosis was carried out according to the radiographic reports of upper urinary tract imaging, including computed tomography (CT), magnetic resonance imaging (MRI), intravenous pyelography, or renal ultrasonography. If more than one imaging modality was available for the same patient, preference was given to the CT report. Overall survival (OS) and cancer-specific survival (CSS) were estimated as the time from RNU to death.

### Follow-up

All patients were generally followed every 3 months in the first year after surgery, then every 6 months from the second to the fifth years, and annually thereafter. Routine surveillance protocol included cystoscopy and blood/urine tests. Urinary cytology, elective bone scans, abdominal or chest CT, and MRI were performed when clinically indicated.

### Statistical Analysis

All statistical analyses were performed using PASW Statistics 18.0 (IBM Corp., NY, USA). Continuous variables were presented as median (interquartile range [IQR]) and compared using the Mann-Whitney U test. Categorical variables were presented as frequency (percentage) and compared using the chi-square test. Survival was compared between patients with or without hydronephrosis using the Kaplan-Meier method and the log-rank test. Multivariable logistic regression and Cox proportional hazard models were used to evaluate the factors associated with pathologic parameters and survival. The variables that were significant in univariable analyses were entered into the multivariable analysis. P <0.05 was considered statistically significant.

## RESULTS

A total of 377 patients were included in the study. The clinical characteristics of the patients are summarized in Table-1. The median follow-up was 36 months (IQR: 20-48 months). Low-grade cancers were detected in 151 (40.1%) patients, whereas 226 (59.9%) patients had high-grade cancers. In addition, 212 (56.2%) patients had preoperative hydronephrosis, including 60 (34.1%) out of 176 patients with renal pelvic tumor, 121 (80.7%) out of 150 patients with ureteral tumor, and 31 (60.8%) out of 51 patients with multifocal tumor.

**Table 1:** Clinical characteristics of patients with upper tract urothelial carcinoma.

Variable		Patients (n=377)
Age (years), median (interquartile range)		70 (60-76)
Male, n (%)		247 (65.5)
**Tumor side, n (%)**		
	Left	211 (56.0)
	Right	166 (44.0)
**Tumor size (cm), n (%)**		
	≤2.5	149 (39.5)
	>2.5	228 (60.5)
**Tumor location, n (%)**		
	Renal pelvis	176 (46.7)
	Ureter	150 (39.8)
	Multifocal	51 (13.5)
Previous or current NMIBC, n (%)		58 (15.4)
Hypertension or diabetes mellitus, n (%)		170 (45.1)
Hydronephrosis, n (%)		212 (56.2)
**pT stage, n (%)**		
	pTa and pT1	149 (39.5)
	pT2	87 (23.1)
	pT3	125 (33.2)
	pT4	16 (4.2)
**Tumor grade, n (%)**		
	Low	151 (40.1)
	High	226 (59.9)
**Lymph node involvement, n (%)**		
	pN0/pNx	350 (92.8)
	pN1/2	27 (7.2)
Lymphovascular invasion, n (%)		29 (7.7)
**Type of surgery, n (%)**		
	Open	254 (67.4)
	Laparoscopic	123 (32.6)

**NMIBC** = non-muscle-invasive bladder cancer.

Table-2 presented the clinical characteristics of patients with high-grade UTUC. The median age was 69 years (IQR: 58-76 years). Among the 226 patients with high-grade UTUC, 132 (58.4%) had preoperative hydronephrosis. The proportions of female patients, patients with ureteral tumors, advanced tumor stage (pT3-4), and LNI were significantly higher in the hydronephrosis group than in the non-hydronephrosis group (P=0.002, P <0.001, P <0.001, and P=0.003, respectively).

**Table 2:** Clinical characteristics of patients with high-grade upper tract urothelial carcinoma.

Variable		All (n=226)	No hydronephrosis (n=94)	Hydronephrosis (n=132)	P
Age (years), median (IQR)		69 (58-76)	69 (58-75)	69 (59-76)	0.226
Male, n (%)		147 (65.0)	72 (76.6)	75 (56.8)	0.002
**Tumor side, n (%)**					**0.337**
	Left	131 (58.0)	58 (61.7)	73 (55.3)	
	Right	95 (42.0)	36 (38.3)	59 (44.7)	
**Tumor size (cm), n (%)**					**0.064**
	≤2.5	85 (37.6)	42 (44.7)	43 (32.6)	
	>2.5	141 (62.4)	52 (55.3)	89 (67.4)	
**Tumor location, n (%)**					**<0.001**
	Renal pelvis	101 (44.7)	64 (68.1)	37 (28.0)	
	Ureter	95 (42.0)	18 (19.1)	77 (58.3)	
	Multifocal	30 (13.3)	12 (12.8)	18 (13.6)	
Previous or current NMIBC, n (%)		33 (14.6)	10 (10.6)	23 (17.4)	0.154
Hypertension or diabetes mellitus, n (%)		98 (43.3)	38 (40.4)	60 (45.5)	0.452
**pT stage, n (%)**					**<0.001**
	pTa and pT1	81 (35.8)	53 (56.4)	28 (21.2)	
	pT2	42 (18.6)	7 (7.4)	35 (26.5)	
	pT3	90 (39.8)	31 (33.0)	59 (44.7)	
	pT4	13 (5.7)	3 (3.2)	10 (7.6)	
**Lymph node involvement, n (%)**					**0.003**
	pN0/pNx	206 (91.2)	92 (97.9)	114 (86.4)	
	pN1/2	20 (8.8)	2 (2.1)	18 (13.6)	
	Lymphovascular invasion, n (%)	22 (9.7)	6 (6.4)	16 (12.1)	0.151
**Type of surgery, n (%)**					**0.488**
	Open	143 (63.3)	57 (60.6)	86 (65.2)	
	Laparoscopic	83 (36.7)	37 (39.4)	46 (34.8)	

**IQR** = interquartile range; **NMIBC** = non-muscle-invasive bladder cancer.

The multivariable logistic regression analyses showed that preoperative hydronephrosis was the only factor that was significantly associated with advanced pT stage in patients with high-grade UTUC (odds ratio [OR]=1.933, 95% confidence interval [CI]: 1.124-3.323, P=0.017). Preoperative hydronephrosis (OR=3.786, 95%CI: 1.617-8.867, P=0.002) and female gender (OR=0.400, 95%CI: 0.174-0.918, P=0.031) were independently associated with LNI or LVI (Table-3). In contrast, preoperative hydronephrosis was not significantly associated with an advanced pT stage (OR=1.038, 95%CI: 0.404-2.664, P=0.938) or LNI/LVI (OR=3.875, 95%CI: 0.788-19.048, P=0.095) in patients with low-grade UTUC. Only the tumor location (ureter vs. renal pelvis) was associated with an advanced pT stage (OR=0.055, 95%CI: 0.012-0.251, P <0.001). Only the tumor side (right vs. left) was associated with LNI/LVI (OR=0.196, 95%CI: 0.039-0.983, P=0.048).

**Table 3:** Multivariable logistic regression analysis for adverse pathologic features in patients with high-grade upper tract urothelial carcinoma.

Variable		High pT stage (pT3-4)		Lymph node involvement and/or lymphovascular invasion	
		OR (95% CI)	P	OR (95% CI)	P
Age		0.758 (0.434-1.323)	0.33	0.993 (0.468-2.105)	0.985
Gender (female vs. male)		0.742 (0.412-1.335)	0.319	0.400 (0.174-0.918)	0.031
Tumor side (right vs. left)		1.139 (0.656-1.977)	0.644	0.947 (0.448-2)	0.886
Tumor size (>2.5 vs. ≤2.5cm)		1.573 (0.881-2.809)	0.125	1.337 (0.593-3.017)	0.484
**Tumor location**					
	Ureter vs. renal pelvis	1.007 (0.52-1.948)	0.985	0.758 (0.321-1.791)	0.528
	Multifocal vs. renal pelvis	0.689 (0.284-1.673)	0.410	0.366 (0.092-1.463)	0.155
Previous or current NMIBC (yes vs. no)		1.229 (0.555-2.718)	0.611	1.463 (0.535-4.001)	0.458
Hydronephrosis (yes vs. no)		1.933 (1.124-3.323)	0.017	3.786 (1.617-8.867)	0.002
Hypertension or diabetes mellitus (yes vs. no)		1.264 (0.725-2.206)	0.409	1.257 (0.595-2.655)	0.548

**NMIBC** = non-muscle-invasive bladder cancer; **OR** = odds ratio; **CI** = confidence interval.

**Figure 1:**
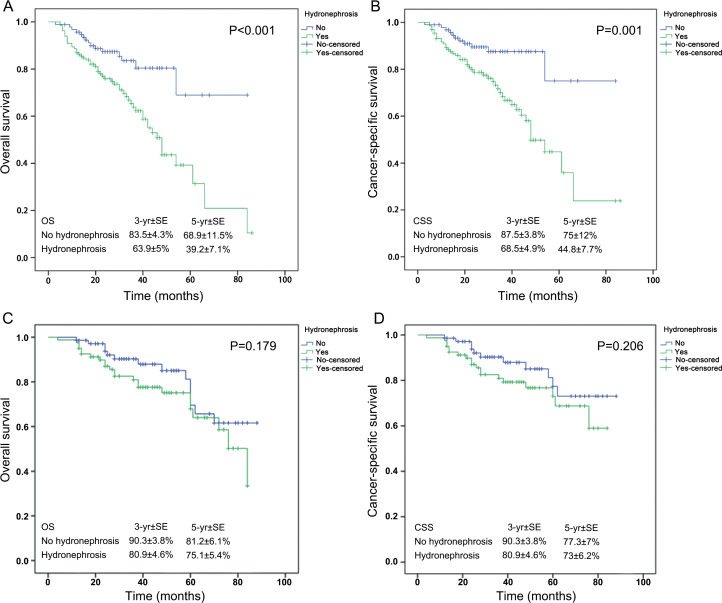
Survival of patients with UTUC. (A) Overall survival in 226 patients with high-grade UTUC. (B) Cancer-specific survival in 226 patients with high-grade UTUC. (C) Overall survival in 151 patients with low-grade UTUC. (D) Cancer-specific survival in 151 patients with low-grade UTUC. CSS, cancer-specific survival; OS, overall survival; SE, standard error; UTUC, upper tract urothelial carcinoma.

During follow-up, in the high-grade group, 67 (29.6%) patients died, including 54 (23.9%) from UTUC. The 3- and 5-year OS rates in patients with hydronephrosis were significantly lower than in patients without hydronephrosis (63.9% and 39.2% vs. 83.5% and 68.9%, respectively; P <0.001) (Figure-1A). The 3- and 5-year CSS rates in patients with hydronephrosis were significantly lower than in patients without hydronephrosis (68.5% and 44.8% vs. 87.5% and 75%, respectively; P=0.001) (Figure-1B). The multivariable Cox regression analyses showed that age (hazard ratio [HR]=2.220, 95%CI: 1.346-3.662, P=0.002), hydronephrosis (HR=2.615, 95%CI: 1.468-4.658, P=0.001), and pT stage (pT1/pTa: HR=1; pT2: HR=4.169, 95%CI: 1.617-10.749, P=0.003; pT3: HR=4.954, 95%CI: 2.054-11.947, P <0.001; pT4: HR=7.073, 95%CI: 2.470-20.247, P <0.001) were independently associated with OS in high-grade disease. Age (HR=1.829, 95%CI: 1.060-3.156, P=0.030), hydronephrosis (HR=2.665, 95%CI: 1.361-5.221, P=0.004), pT stage (pT1/pTa: HR=1; pT2: HR=4.567, 95%CI: 1.459-14.295, P=0.009; pT3: HR=5.767, 95%CI: 1.992-16.697, P=0.001; pT4: HR=9.027, 95%CI: 2.661-30.621, P <0.001), and LNI (HR=2.239, 95%CI: 1.174-4.272, P=0.014) were independently associated with CSS in high-grade disease. In the low-grade cohort, hydronephrosis was not associated with postoperative survival (Figures 1C and D).

## DISCUSSION

In accordance with previous studies (20-22), pathologic features such as pT, LNI, and LVI were confirmed to be the most crucial prognostic factors for UTUC in this study. In addition, we surprisingly found that hydronephrosis was significantly associated with an advanced pT stage (P=0.017) and LNI or LVI (P=0.002) in the high-grade UTUC group (Table-3), thereby demonstrating a surrogate associated factor of adverse pathologic features in UTUC.

In the present study, higher frequency of preoperative hydronephrosis was observed in patients with ureteral tumor than in those with pelvic tumor, both in high-grade (81.0% [77/95] vs. 36.6% [37/101]) and low-grade (80.0% [44/55] vs. 30.7% [23/75]) UTUC groups. Ureteral tumors might be faster to produce hydronephrosis than pelvic tumors., Indeed it has been reported that ureteral tumors were more likely to present hydronephrosis than renal pelvic tumors (13). Previous series also showed that ureteral tumors were associated with hydronephrosis, while pelvic tumors were not associated, except tumors of the pelviureteric junction (11, 15, 23, 24). Furthermore, the pattern of invasion of the surrounding tissues might affect the development of hydronephrosis. Pelvic tumors will invade into the renal parenchyma and the perinephric fat, but the likelihood of causing obstruction is small; on the other hand, ureteral tumors will directly invade the peri-ureter tissues, with a higher likelihood of compressing the ureter and obstructing urine flow (25).

Several studies attempted to investigate hydronephrosis in the prediction of pathologic features of cancers of the bladder and upper urinary tract. In patients with bladder cancer, Stimson et al. (26) reported that the presence of hydronephrosis before radical cystectomy was an independent predictor of adverse pathologic features (extravesical and node-positive disease). Small-sample studies reported a similar association, i.e., that hydronephrosis was significantly associated with muscle-invasive or non-organ-confined disease (11, 15). Afterward, Messer et al. (12) and Chung et al. (18) also reported this association, respectively, in larger multicenter studies of patients with high-grade UTUC. Nevertheless, conclusions from previous studies are often limited by the small samples (<150 patients). In addition, although the study by Messer et al. had a larger cohort, the lack of oncologic follow-up and the enrollment of patients who only underwent distal ureterectomy (9%) biased their results. Therefore, conducting a study on the prediction potential of hydronephrosis in UTUC was of clinical significance.

Different from most previous studies on the methodological point of view, the present study investigated the pathologic and prognostic relevance of hydronephrosis by subgroup analysis in a cohort of patients with high-grade UTUC. The association with muscle-invasive or non-organ-confined UTUC implies that hydronephrosis is likely caused by luminal obstruction as well as intramural invasion or extrinsic compression. In contrast, hydronephrosis may cause outward expansion and longitudinal thinning of the upper urinary tract, facilitating the seeding of cancer cells to regional or distant organs. Compared with high-grade disease, a low-grade disease is less poorly differentiated and less aggressive. Thus, the expanding pressure of hydronephrosis could less likely result in the spreading or aggressiveness of the disease in patients with low-grade UTUC. This indicates that analyzing all UTUCs together might dilute the pathologic and prognostic relevance of hydronephrosis. The subgroup analysis in the low-grade group demonstrated that hydronephrosis was not significantly associated with adverse pathologic features.

Notwithstanding, the prognostic relevance of hydronephrosis in UTUC after RNU still remains a controversial issue. In this study, according to the multivariable analysis, after incorporating only preoperative factors, hydronephrosis was found to be independently associated with OS and CSS (P=0.001 and P=0.004, respectively) in the high-grade cohort, which is in line with several previous studies. This is supported by available prognostic models and nomograms for UTUC; those that include hydronephrosis have a higher accuracy (27). Zhang et al. found that preoperative hydronephrosis was an independent predictor for CSS and progression-free survival (P=0.001 and P=0.007, respectively). Therefore, they reported that ureteral tumors showed a worse prognosis than renal pelvis tumors, which is likely due, at least in part, to more frequent hydronephrosis (13). Similarly, in another study, Ng et al. demonstrated that hydronephrosis was associated with worse CSS and metastasis-free survival when only preoperative variables were controlled (P=0.001 and P=0.004, respectively) (11). On the other hand, the studies conducted by Bozzini et al. (16) and Ito et al. (15) failed to show any difference in outcomes between the groups stratified by hydronephrosis. Thus, owing to the heterogeneity in methodology and based on the different criteria for hydronephrosis assessment and survival analysis, performing a comparative analysis among these studies would be fruitless. Nevertheless, we believe that preoperative hydronephrosis should be included in the evaluation of prognosis when it is taken into account as simple radiographic comorbidity and surrogate for poor pathologic features in patients with high-grade UTUC.

There are several mechanisms that might explain why hydronephrosis is an independent predictive factor for OS and CSS in the present study. First, Zhang et al. (13) reported that ureteral tumors were more likely to present hydronephrosis and less likely to have hematuria compared with renal pelvic tumors. Thus, it is reasonable to believe that ureteral tumors are asymptomatic during the early stages because of incomplete obstruction, and presenting with hydronephrosis perhaps only during the late stages. The development of hydronephrosis might need some time after urinary tract obstruction resulting from the tumor. As a result, UTUC might progress during the development of hydronephrosis (13). Second, several studies reported that patients with hydronephrosis had a more advanced tumor stage than those without hydronephrosis (13, 14, 28). In addition, ureteral tumors were reported to be more likely to present with hydronephrosis and to have a worse prognosis than renal pelvic tumors, which could be attributed to the thin layer of surrounding ureteral adventitia containing an extensive plexus of blood vessels and lymphatic channels, making tumor invasion and metastasis easier (29, 30). The exact reasons why hydronephrosis is associated with an adverse prognosis in high-grade UTUC still need to be determined.

We must emphasize the limitations of this study. First and foremost, the retrospective nature of the study resulted in an accumulation of variability and inherent biases. Especially, the glomerular filtration rate could not be analyzed because of the amount of missing data. Second, the radical surgeries were performed by several surgeons, possibly resulting in some variations among cases. Third, although the CT or MRI images showed that all patients had renal pelvis or calyx dilation with proximal ureteral dilatation, we could not review the ultrasound images because our hospital does not retain ultrasound images. A previous study demonstrated that ultrasound and CT scans had different sensitivity, specificity, positive likelihood ratio, and negative likelihood ratio for the diagnosis of hydronephrosis (31). This might be a limitation because some patients were diagnosed only by ultrasound. Fourth, it would provide greater information if we could evaluate an association between hydronephrosis and disease recurrence or progression. Fifth, patients who received perioperative chemotherapy were excluded because chemotherapy could influence survival, and to reduce the heterogeneity of the study population. On the other hand, we could not examine the influence of perioperative chemotherapy on the association between hydronephrosis and OS/CSS. Finally, because of the retrospective nature of the study, we were limited to the data available in the medical charts. Future studies could look at the combination of clinical markers combined with tissue-based molecular markers and inflammation scores to construct predictive and prognostic models for UTUC (32, 33). Overall, the surprising predictive value of preoperative hydronephrosis regarding pathology and prognosis of UTUC might be clinically significant.

## CONCLUSIONS

In conclusion, preoperative hydronephrosis was associated with adverse pathologic features and postoperative survival in patients with high-grade UTUC. This finding suggests that hydronephrosis, as a promising prognostic factor, could be precious in properly guiding therapeutic approaches such as neoadjuvant chemotherapy and lymphadenectomy.

## ABBREVIATIONS

CSS: cancer-specific survival
CI: confidence interval
CT: computed tomography
HR: hazard ratio
IQR: interquartile range
LNI: lymph node involvement
LVI: lymphovascular invasion
MRI: magnetic resonance imaging
NMIBC: non-muscle invasive bladder cancer
OR: odds ratio
OS: overall survival
pT: primary tumor stage
RNU: radical nephroureterectomy
UTUC: upper tract urothelial carcinoma
